# Attraction to Carbon Dioxide from Feeding Resources and Conspecific Neighbours in Larvae of the Rhinoceros Beetle *Trypoxylus dichotomus*

**DOI:** 10.1371/journal.pone.0141733

**Published:** 2015-11-04

**Authors:** Wataru Kojima

**Affiliations:** Graduate School of Arts and Sciences, The University of Tokyo, 3-8-1 Komaba, Meguro-ku, Tokyo, 153-8902, Japan; University of Tours, FRANCE

## Abstract

Saprophagous (feeding on decaying matter) insects often use carbon dioxide (CO_2_) as a cue for finding food. Humus-feeding larvae of the giant rhinoceros beetle *Trypoxylus dichotomus* exhibit a clumped distribution in natural microhabitats, but the mechanisms driving the distribution were unknown. Herein, I examined whether larvae use CO_2_ as a cue for fermented humus and aggregate in the vicinity of the food. I found that (i) larvae of *T*. *dichotomus* are strongly attracted to CO_2_, (ii) larvae orient toward highly fermented humus when given a choice between highly and poorly fermented humus, (iii) the highly fermented humus emits more CO_2_ than the poorly fermented humus, and (iv) larvae grow larger when fed highly fermented humus rather than poorly fermented humus. The clumped distribution of larvae is probably formed along the concentration gradient of CO_2_ induced by heterogeneity of fermented organic materials in soil. My laboratory experiments also revealed that larvae are chemically attracted to each other. Moreover, CO_2_ concentrations in soil were increased by the larval respiration, and small amounts of CO_2_ (much less than emitted during respiration by a single larva) were sufficient for larval attraction. These results suggest that not only response to fermented food resources, but also respiratory CO_2_ from conspecifics may lead to aggregation. Enhanced densities resulted in reduced weight gain under experimental conditions. However, exploiting a high-value resource at enhanced densities still led to greater body weight compared to individually exploiting a low-value resource. This demonstrates the adaptive value of the response to CO_2_ sources in this species.

## Introduction

Within a highly complex environment, insects need to locate their potential food resources. Chemical cues play a crucial role in finding distant resources. Especially for soil-living insects that cannot rely on visual cues, a broad range of chemical cues is used [1–3]. Among them, carbon dioxide (CO_2_) elicits a strong behavioural response in the majority of soil-living insects including phytophagous [1–6] and saprophagous species [7], [8]. Because CO_2_ is emitted from various sources other than food, the response of insects to CO_2_ is sometimes modulated by additional specific cues [1–3]. For example, larvae of the root-feeding weevil *Sitona lepidus* are not attracted to CO_2_, but initiate intensive searching behaviour to locate more specific chemical cues to their host in the presence of CO_2_ [4]. On the other hand, CO_2_ has been reported to represent a sufficient orientation cue for many species [9], [10]. Thus, they may be attracted to CO_2_ sources other than their food under natural conditions, which in turn may affect insect feeding and performance.

**Fig 1:**
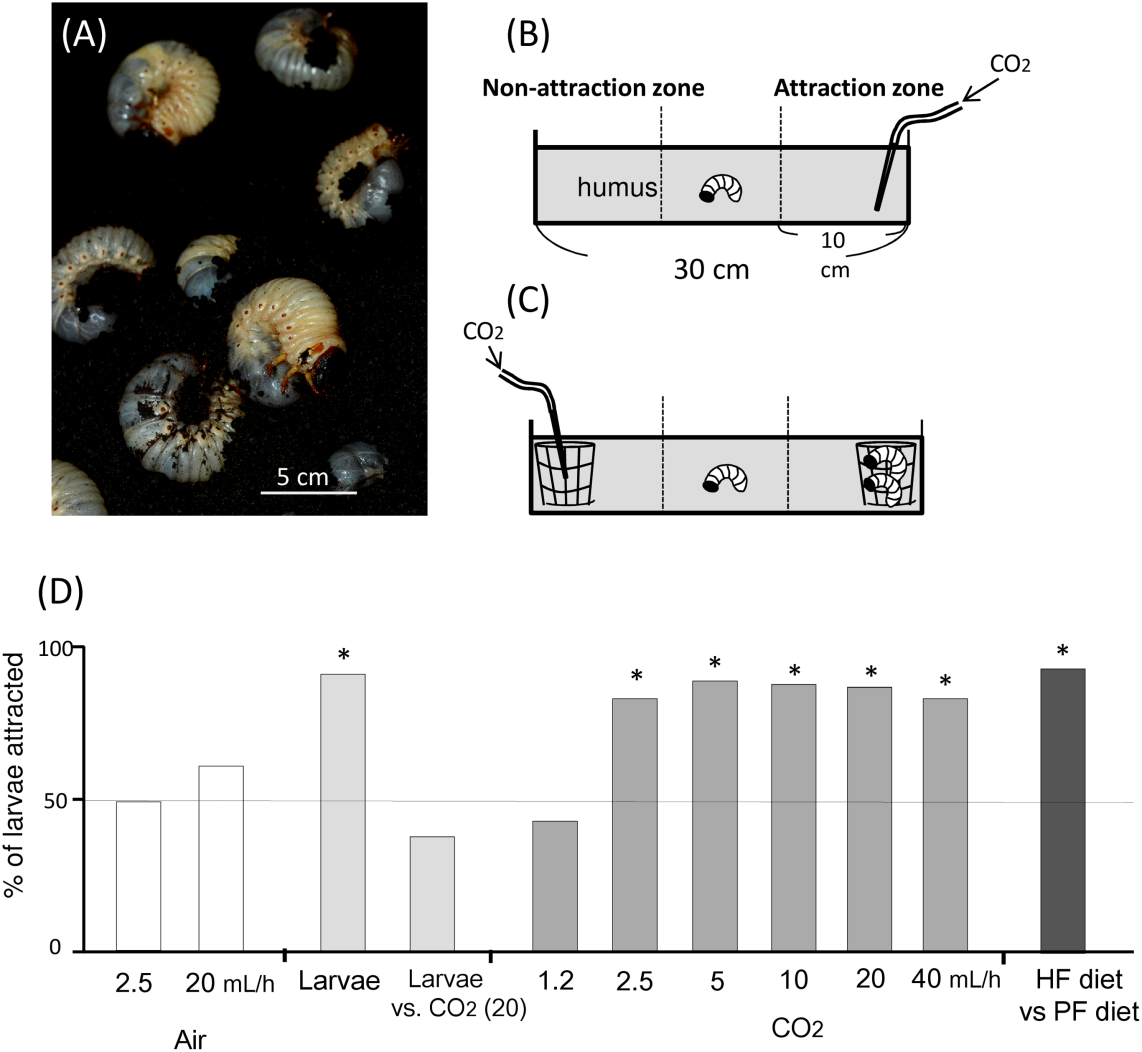
*Trypoxylus dichotomus* larval response to humus, conspecifics and synthetic CO_2_ (A) A group of 3rd instar larvae in a microhabitat. Soil above the larvae was removed. (B and C) Schematic representation of the behavioural arena established in a previous study [11]. In (B), the larval response to various amounts of CO_2_ (or air for the control) was tested. CO_2_ was pumped through a pipette into the soil using a syringe driver. In (C), larvae were given the choice of 20 mL/h of CO_2_ or two conspecific larvae in a mesh cage. In both systems, ‘attraction zones’ and ‘non-attraction zones’ were set within 10 cm from each end. (D) The percentage of larvae attracted to air, CO_2_, highly fermented (HF) humus [against poorly fermented (PF) humus] or to two larvae in a mesh cage. The total number of larvae found in the attraction zone was compared with that of larvae in the non-attraction zone. Asterisks indicate a significant difference (*P* < 0.05) from 50% (dashed line); analysed using the binomial test. The sample sizes (the sum of the larvae in the attraction and non-attraction zones) were 12, 17, 22, 20, 20, 22, 19, 16, 20, 15, and 16 from left to right.

Larvae of the giant rhinoceros beetle *Trypoxylus dichotomus* inhabit the soil humus layer and feed on decaying organic matter. The larvae are known to exhibit a clumped distribution throughout their larval periods in natural humus microhabitats (Fig 1A) [11]. This clumped distribution is likely because of clumped deposition of eggs and heterogeneity of humus [11], but the mechanism driving the formation of the distribution remains unclear. I hypothesized that the larvae aggregate in a zone of high saprophytic activity using chemical cues, such as CO_2_ from microbial respiration. Furthermore, it has been suggested that the larvae of *T*. *dichotomus* are attracted to chemicals from conspecifics [11]. The chemicals have not been identified, but respiratory CO_2_ may be involved in conspecific attraction if larvae use CO_2_ to find food resources. Herein, I first examined whether the larvae were attracted to CO_2_ from food resources and conspecifics. Second, I examined whether the aggregation leads to decreased or increased larval performance in terms of weight gain. Although some group-living insects may gain net benefits by group formation [12–14], aggregation often increases competition for resources, negatively affecting performance [15], [16].

## Materials and Methods

All the experiments and insect rearing were conducted at 24.5–25.0°C and 60–70% relative humidity. The air in the laboratory was constantly exchanged using an air fan, and the concentration of CO_2_ was maintained at 420–450 ppm. The humus used was Sanyo-Bark (Sanyo Chip Kogyo Co. Ltd., Yamaguchi, Japan). However, in rearing experiments and the choice test between two types of humus, less-fermented bark that was sold for stag beetle rearing (Dorcus Owner’s Shop, Osaka, Japan) and fermented bark commercially sold for rearing of beetles belonging to the family Dynastinae (Dorcus Owner’s Shop) was used.

### Insects

The *Trypoxylus dichotomus* larvae used in all experiments, except for the rearing experiments, were in the middle of their 3rd (final) instar (70–110 days old). These were individuals from a colony established from adults (*n* = 13) that had been collected from several fields in Tokyo during the summers of 2012 and 2013. *T*. *dichotomus* is not an endangered or protected species, and collection of unprotected insects in this area does not require special permission. Further details of the egg collection and larval rearing methods are available in Kojima et al. [11]. Briefly, females were individually reared in cages containing humus. Their eggs were collected once a week and individually introduced into 450-mL plastic cups filled with humus. The humus was renewed every 20–30 days. The body weight of the larvae used in the experiments was between 18–25 g. For rearing experiments, I used early-stage 2nd instar larvae (1.0–1.5 g).

### Measurement of larval and soil respiration

I measured the emission rate of respiratory CO_2_ of *T*. *dichotomus* larvae and two types of humus used in the choice test and rearing experiments using a Vaisala CARBOCAP Hand-Held Carbon Dioxide Meter GM70 with a GMP222 infrared CO_2_ probe (Vaisala Oyj, Helsinki, Finland). The accuracy of the probes was ±2% of the reading. The system was equilibrated for 5 min prior to measuring the next sample.

A 3rd instar larva and a small electric fan were placed inside an 860-mL plastic cup, and a CO_2_ probe was inserted through a hole in the lid of the cup. To measure humus respiration, 80 g of humus soil was placed into a sealed plastic chamber (1.7 L) with the fan. I filled any gaps with paper clay to avoid exposure to air. The CO_2_ concentration in the cup or chamber linearly increased immediately after the introduction of a larva or humus. Immediately after and for 5 min after the introduction of a larva or humus, the CO_2_ concentration in the cup or chamber was recorded. CO_2_ production rate (nmol min^-1^ g^-1^) of a lava or humus was calculated from the concentration increments during 5 min. The measurement was replicated using 6 different samples or larvae.

### Larval response to synthetic CO_2_ and humus

I investigated the responses of *T*. *dichotomus* larvae to conspecifics, synthetic CO_2_ and humus to clarify the the mechanism driving the formation of the larval clumped distribution. The experimental system, following the method described by Kojima et al. [11], is shown in Fig 1B and 1C.

Firstly, I tested whether *T*. *dichotomus* larvae were attracted to conspecifics. I buried two steel-wire screen cages (6 cm diameter × 7 cm height; mesh size, 3 mm), one containing only humus (Sanyo-Bark) and the other containing humus and two 3rd instar *T*. *dichotomus* larvae, at the ends of a rectangular plastic container (30 cm × 7 cm × 7 cm) filled with humus. I then placed the experimental larva at the centre of the container, and allowed it burrow into the humus for 1 h. The position of the larva’s head was recorded at the end of each experiment. Larvae found within 5 cm of the release point were excluded from data analysis (<20% in all the behavioural assays). The total number of larvae found in the attraction zone (n_1_) was compared with that in the non-attraction zone (n_2_). The null hypothesis, n_1_/(n_1_ + n_2_) = 0.5, was tested using the binomial test.

I tested whether *T*. *dichotomus* larvae were attracted to synthetic CO_2_. A 25-mL syringe was filled with CO_2_ (>99.5% purity) and connected to a silicon tube that was attached to a Pasteur pipette. CO_2_ was constantly pumped through the pipette using a syringe pump (Sanyo Chip Kogyo Co. Ltd., Kyoto, Japan), at a rate of 1.2, 2.5, 5, 10, 20, or 40 mL h^-1^. The pipette was buried in humus at the end of the rectangular cage, as shown in Fig 1B. I placed the experimental larva at the centre of the container and recorded the position of the larva’s head 1 h later. As a control, air instead of CO_2_ was provided at a rate of 2.5 or 20 mL h^-1^.

I tested whether *T*. *dichotomus* larvae prefer conspecifics to CO_2_, using the experimental system shown in Fig 1C. Two steel-wire screen cages containing only humus or humus and two 3rd instar *T*. *dichotomus* larvae were buried at opposite ends of the rectangular container. In the screen cage without insects, CO_2_ was provided through a Pasteur pipette at a rate of 20 mL h^-1^ (corresponding to the respiration of two larvae), as described above.

Furthermore, I tested if larvae preferred highly fermented humus to poorly fermented humus. Two steel-wire screen cages containing high-quality humus or low-quality humus (ca. 80 g) were buried at the opposite ends of the rectangular container. In this test, black dirt (Ratec, Gunma, Japan) was used as a substrate instead of humus. My preliminary experiment showed that black dirt emits only a small amount of CO_2_ (< 0.5 nmol min^-1^ g^-1^).

The humus soil in the rectangular container was stirred and the two ends were exchanged after each trial. The soil was renewed after 5 successive trials. Sample sizes are presented in the Fig 1D legends.

### Effect of larval density on CO_2_ concentration

I determined if larvae increased CO_2_ concentration in humus. I randomly placed 24 *T*. *dichotomus* larvae into a plastic cage (44 cm × 66 cm × 40 cm in height) that contained humus to a depth of 30 cm. The larval density corresponded with that observed within natural microhabitats [11]. Another cage containing the same amount of humus was used as a control. After five days, the two cages were evenly divided into six squares (22 cm × 22 cm), and the soil CO_2_ concentration at a depth of 20 cm was recorded at the centre of each square using the aforementioned CO_2_ meter, the probe of which was covered with an in-soil adapter (Vaisala). I also measured soil temperature at a depth of 20 cm using a digital thermometer (Sato Keiryoki MFG. Co. Ltd., Tokyo, Japan) because larvae might increase soil temperature by activation of soil microbes. Immediately after measuring the CO_2_ and temperature in all six squares, I removed the humus and counted the number of larvae in each square of the treatment cage (i.e., the cage containing larvae). If larvae were on the border between squares, the position of the head was recorded. Furthermore, soil respiration apart from larval respiration was examined as an index of microbial activity because CO_2_ concentration in humus may be affected by the fermentation level of the humus. Eighty grams of humus soil was collected from the bottom of each square and immediately placed into a sealed plastic chamber (1.7 L). The CO_2_ concentration in the chamber linearly increased. Increments of CO_2_ concentration were measured using the CO_2_ meter for 5 min, and the rate of CO_2_ emission (nmol min^-1^ g^-1^) was calculated as described above. The mean CO_2_ concentration, soil respiration, and temperature for the six squares were calculated for each cage. All humus in the two cages (control and treatment) was renewed prior to the next trial. The trials were replicated eight times in total. A paired *t*-test was used for the comparison between the control and treatment.

I examined the relationship between larval density (i.e., the number of larvae in a square) and the CO_2_ concentration, soil respiration or soil temperature in the treatment cage using a linear mixed model (LMM). The CO_2_ concentration, soil respiration or soil temperature was used as an objective variable, and larval density was used as the explanatory variable. The baseline of CO_2_ concentrations were different among the eight trials (see Results), and thus, trial identity was included as a random factor in order to account for the variance. These analyses were performed using the statistical platform R v. 3.0.1 [17].

### Effects of larval density and humus quality on body weight gain

I investigated whether (i) larvae grow larger when fed highly fermented rather than poorly fermented humus and (ii) larvae incur a fitness cost or benefit in terms of body weight gain when they aggregate. For this experiment, the larvae had been individually reared after hatching in fermented bark (highly-quality food). The 2nd instar larvae (1.0–1.5 g) were reared under solitary, medium-density (three larvae), and high-density (nine larvae) conditions for 5 days in 860 mL plastic cups. Larval heads were painted with an oil-based ink for identification. The cup was filled with highly fermented or poorly fermented humus. Larval body weight was measured before and after 5 days of treatment. The larvae were individually reared for a month after the treatment and their sex was determined at the 3rd instar [18]. The effects of food quality and density on larval weight after 5 days of treatment were determined using a LMM. The identity of the mother of each larva was included as a random factor because a previous study indicated that offspring body size varies depending on maternal identity [19]. Body weight after treatment was a response variable, and density, food quality, and the interaction between density and food quality were included as explanatory variables. The initial body weight and sex were also included as covariates. Since the interaction term was significant (see Results), post-hoc analyses were conducted under each nutrient condition using a LMM in which density, initial body weight and sex were entered as explanatory variables. Pairwise comparisons between treatments were subsequently conducted using the LMM with Tukey’s tests. The number of larvae used for each treatment was 26–41.

## Results

### Measurement of larval and soil respiration

The larvae of *T*. *dichotomus* emitted 356 ± 15 nmol min^-1^ g^-1^ (mean ± SE, *n* = 6) of CO_2_. The highly fermented and poorly fermented humus emitted 9.00 ± 0.23 (*n* = 6) and 2.09 ± 0.10 nmol min^-1^ g^-1^ (*n* = 6) of CO_2_, respectively.

### Larval response to synthetic CO_2_ and humus

In the behavioural assays using two caged larvae, the frequency of larvae found was significantly greater in the attraction zone than in the non-attraction zone (*P* < 0.01 by the binomial test, Fig 1D). When various amounts of CO_2_ (1.2, 2.5, 5, 10, 20, or 40 mL h^-1^) were provided, larvae of *T*. *dichotomus* were attracted to 2.5–40 mL/h of CO_2_ within 1 h (Fig 1D). The larvae were not attracted to air (Fig 1D). When two caged larvae were tested against 20 mL h^-1^ of CO_2_, no significant difference was observed between the two choices (larvae vs. CO_2_; Fig 1D). In the choice between highly and poorly fermented humus, larvae were attracted to highly fermented humus (Fig 1D).

### Effect of larval density on CO_2_ concentration

**Fig 2:**
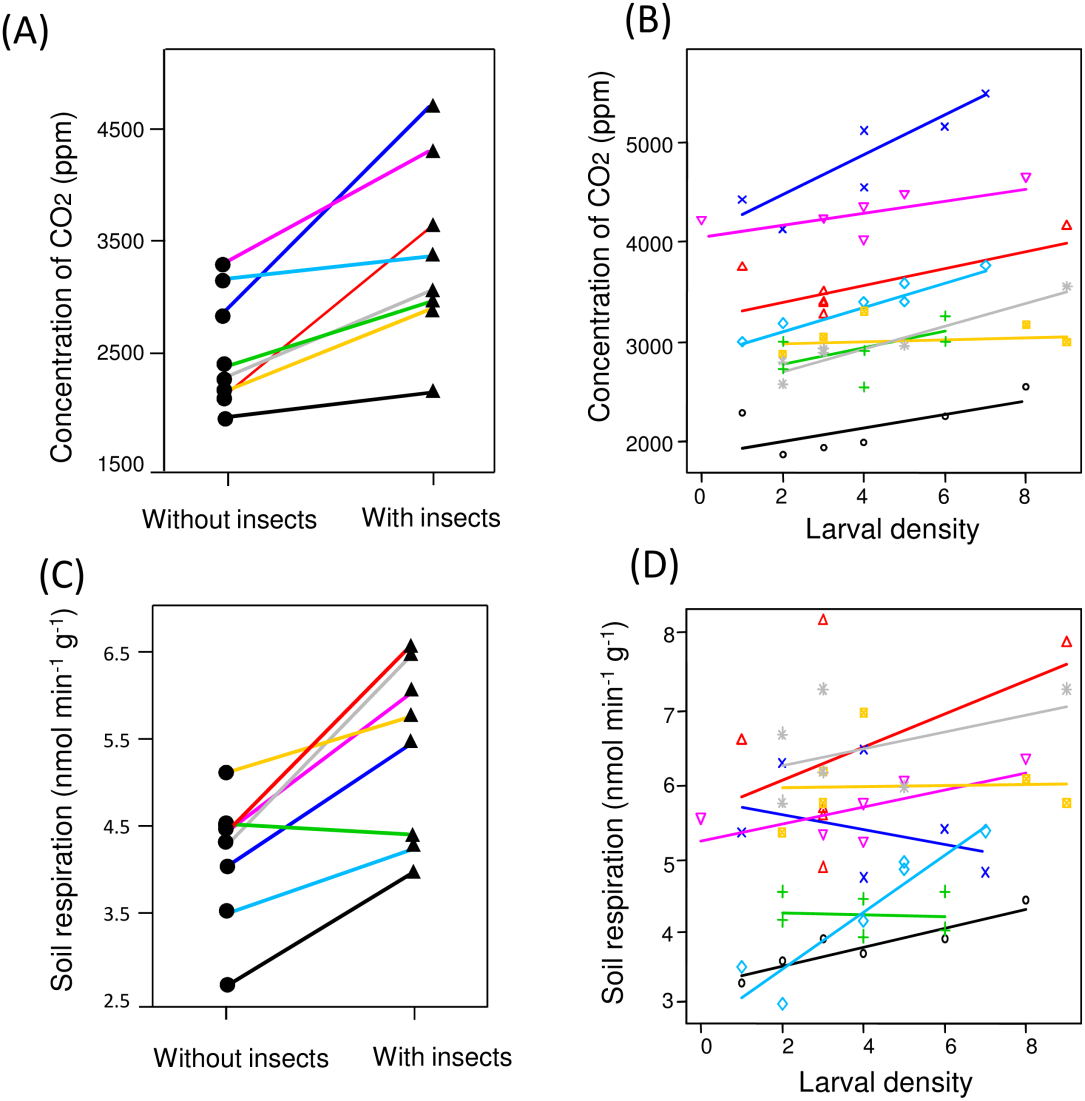
CO_2_ concentration and *Trypoxylus dichotomus* larval density/presence. (A) Comparison of soil CO_2_ concentrations between containers (triangles) with and without insects (circles). The data from eight pairs of replicates are presented in different colours. (B) The relationship between larval density (i.e., the number of larvae in a quadrat) and CO_2_ concentration in that quadrat. (C) Comparison of soil respiration between containers with (triangles) and without insects (circles). (D) The relationship between larval density and soil respiration.

The soil CO_2_ concentration was 32 ± 8.1% (mean ± SE, *n* = 8 cages) higher in cages with insects than in those without insects (Fig 2A; *t*_7_ = 3.82, *P* = 0.0065, paired *t*-test). In cages containing insects, the number of larvae within a square was positively correlated with the soil CO_2_ concentration (Fig 2B; *t* = 4.16, *P* < 0.001, LMM). Soil respiration in cages with insects was 27 ± 6.3% (mean ± SE, *n* = 8 cages) higher than that in cages without insects (Fig 2C; *t*_7_ = 4.13, *P* = 0.0044, paired *t*-test). In cages containing insects, soil respiration rate was higher in the quadrats containing more larvae (Fig 2D; *t* = 3.22, *P* = 0.0018, LMM). Soil temperature was not higher in the squares containing more larvae (*t* = −0.16, *P* = 0.55, LMM), and it was almost significantly higher in cages containing larvae [24.63 ± 0.13°C (mean ± SE, *n* = 8 cages)] than in those without larvae [24.48 ± 0.13°C (mean ± SE, *n* = 8 cages), *t*_7_ = 2.32, *P* = 0.053, paired *t*-test].

### Effect of larval density and humus quality on body weight gain

LMM analysis using pooled data showed that density, food quality, their interaction, sex and initial weight were significantly correlated to larval body weight after treatment (Table 1).

**Table 1:** Larval weight gain under high and low nutrient conditions is density dependent. The effects of density (1, 3, or 9 larvae/cage) and food quality (highly fermented or poorly fermented humus) on larval weight gain were tested using the LMM. Sex and initial weight were included as covariates, and identity of the mother of each larva was included as a random factor. Following an analysis of pooled data, additional analyses under each nutrition condition were conducted because the interaction term between density and food quality was significant.

Explanatory variable	Pooled data		Highly fermented		Poorly fermented	
	*χ* ^2^	*P*	*χ* ^2^	*P*	*χ* ^2^	*P*
Density	47.8	<0.001	29.9	<0.001	28.1	<0.001
Initial weight	89.2	<0.001	40.1	<0.001	35.0	<0.001
Sex	5.06	0.0244	1.8	0.19	10.7	0.001
Food quality	53.1	<0.001	-	-	-	-
Density × Food quality	14.0	<0.001	-	-	-	-

**Fig 3:**
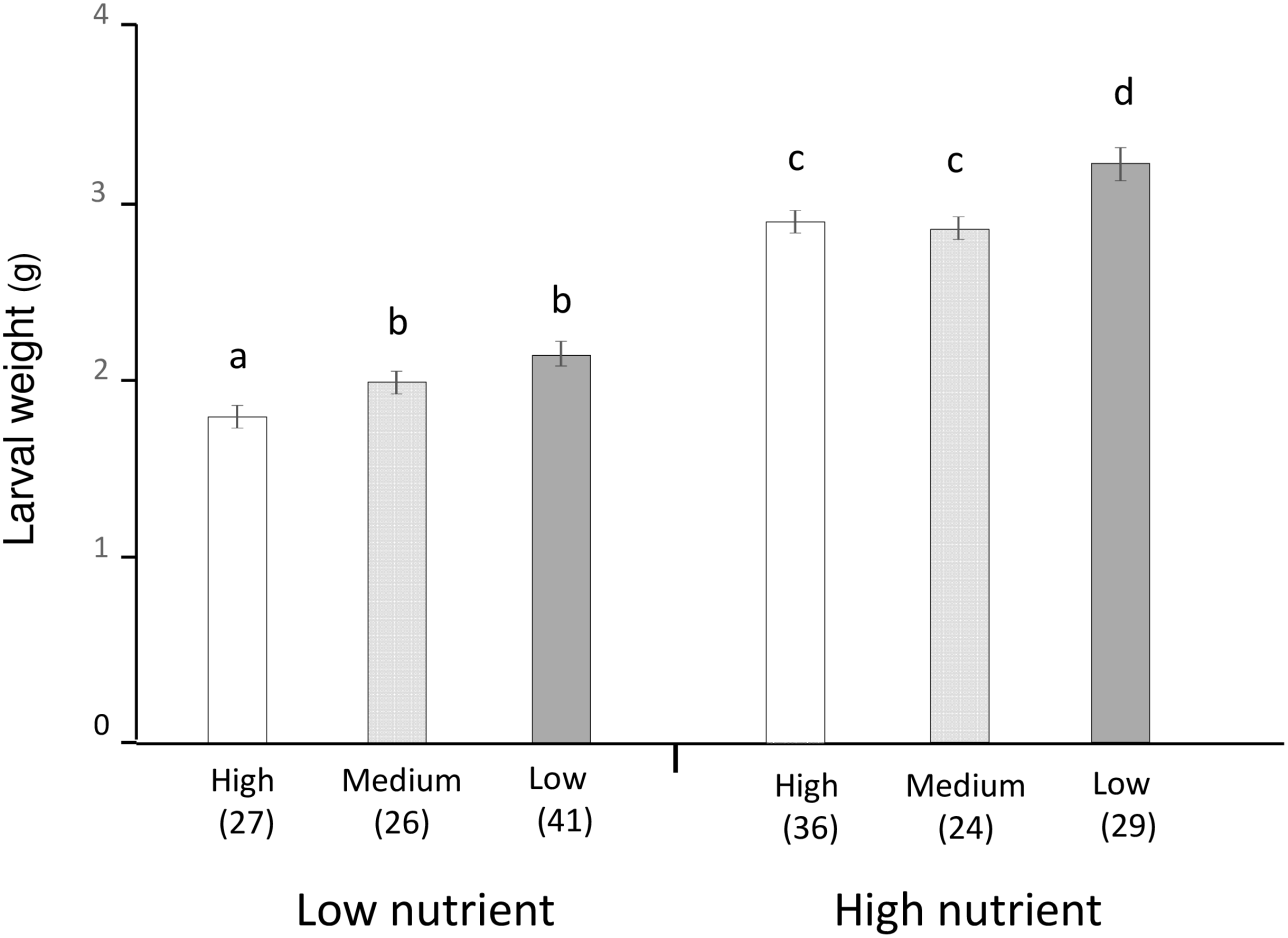
Effect of density on *Trypoxylus dichotomus* larval growth. Body weights of 2nd instar larvae reared at low (1 larva/cage), medium (3 larvae/cage), and high (9 larvae/cage) densities when provided poorly fermented (left) or highly fermented (right) humus. Error bars, SE. Letters indicate *P* < 0.05 in pairwise comparisons analysed by a linear mixed model with Tukey test. Detailed statistical values are presented in Table 1. Sample sizes are shown in parentheses. Error bars, SE.

When larvae were provided poorly fermented food, significant effects of density and initial weight were detected, but sex did not affect body weight after treatment [males: 1.96 ± 0.045 g (mean ± SE, *n* = 51), females: 2.03 ± 0.043 g (*n* = 44)] (Table 1). However, enhancing larval density reduces weight gain under both high and low humus quality. Body weight of larvae reared alone [2.11 ± 0.047 g (*n* = 41)] was 5% and 17% higher than that of larvae reared at medium [2.01 ± 0.049 g (*n* = 26)] or high densities [1.81 ± 0.051 g (*n* = 27)], respectively (Fig 3). Body weight of larvae reared alone and at medium density was significantly greater than that of larvae reared at high densities, but there was no difference between that of larvae reared alone and at medium density (Fig 3).

Under high-nutrient conditions, significant effects of density and initial weight were detected (Table 1). In addition, male larvae were significantly heavier than females [males: 3.02 ± 0.065 g (*n* = 45), females: 2.78 ± 0.045 g (*n* = 44)] (Table 1). The body weight of larvae reared alone [3.14 ± 0.070 g (*n* = 29)] was 15% and 11% higher than that of larvae reared at medium [2.74 ± 0.070 g (*n* = 24)] or high densities [2.82 ± 0.061 g (*n* = 36)], respectively (Fig 3B). The body weight of larvae reared alone was significantly heavier than that of larvae reared at medium and high densities, but there was no difference between that of larvae reared at medium and high densities (Fig 3).

## Discussion

I found that (i) larvae of *T*. *dichotomus* are strongly attracted to CO_2_, (ii) larvae orient toward highly fermented humus when given a choice between highly fermented humus and poorly fermented humus, (iii) highly fermented humus emits more CO_2_ than the poorly fermented humus, and (iv) larvae grow larger when reared in highly fermented rather than poorly fermented humus. Saprophagous insects feed on bacteria or fungi as a main source of protein [20]. CO_2_ is a cue for fermented organic substances in the saprophagous species [7], [8]. The orientation behaviour toward CO_2_ is also widespread in phytophagous [1–6] and blood-sucking species [21], [22], and this behaviour is thought to be an adaptive response for finding food [5].

The larvae of *T*. *dichotomus* are reported to exhibit a clumped distribution within microhabitats [11]. Larval distribution may occur along the concentration gradient of CO_2_. Under natural conditions, the concentration of CO_2_ in soil is probably heterogeneous because of the clumped distribution of fermented resources, and larvae likely aggregate in the vicinity of preferred food. The body weight of larvae reared alone was higher than that of larvae reared at medium or high densities. The high larval density probably leads to intra-specific competition for food and space. This is in contrast to results observed in group-living species in which aggregated individuals show a higher growth rate than solitary individuals [23], [24]. However, the body weight of *T*. *dichotomus* larvae reared at high density in the highly fermented humus was greater than that of larvae reared at low density in the poorly fermented humus. Thus, finding highly fermented humus using CO_2_ is beneficial for larvae even though conspecific neighbours aggregate at the high-CO_2_ site and compete with each other.

Male larvae were heavier than female larvae under high nutrition conditions only. Thus, at this stage a sexual size dimorphism becomes only apparent if high quality food is available. Such sexual difference in plasticity of body size has been reported in other species [25], [26]. Male body size of *T*. *dichotomus* is known to be strongly related to the individual’s abilities in intrasexual competition and male body size may be under positive directional selection [27]. Males may invest as many resources as possible into increasing body growth, which increases the condition dependency (i.e., plasticity) of the trait (see [25]).

Although the heterogeneity of organic materials mainly generates the gradients of CO_2_ concentration in soil, larvae themselves may also partly create the gradients. Consistent with a previous finding [11], larvae were found to be attracted to conspecifics. My laboratory experiments also showed that CO_2_ concentration in soil is increased by the larvae. Moreover, the results of behavioural assays indicated that only a small amount of respiratory CO_2_ (i.e., much less than the CO_2_ emitted during respiration by a single larva) is sufficient for larval attraction. The CO_2_ emission rate of larvae per unit mass was ca. 40- to 100-fold higher than that of substrates. The effect of respiratory CO_2_ on the orientation behaviour of neighbours may not be negligible when heterogeneity of substrates of microhabitats is low.

A previous study showed that conspecific chemical attraction has been observed in 2nd- and 3rd-instars, but not in 1st-instar larvae [11]. Although 2nd and 3rd-instar larvae of this species are large enough to generate steep CO_2_ gradients by respiration within homogeneous soil, small 1st-instar larvae may emit smaller amount of CO_2_ than neighbours are able to detect. The previous study also reported that *T*. *dichotomus* larvae with ablated maxillary palps were not attracted to conspecifics [11]. However, ablating the antennae did not affect the larval aggregation behaviour [11]. Thus, the sensors of CO_2_ in *T*. *dichotomus* appear to be present on maxillary palps but not antennae. The sensory organ for CO_2_ in insects is generally located on maxillary palps (e.g., mosquitoes [28]), labial palps (e.g., moths [29]), or antennae (e.g., fruit flies [30]). In the phytophagous scarab larvae *Melolontha melolontha*, antennae are the only head appendage to physiologically respond to CO_2_, and labial and maxillary palps did not respond to CO_2_ [31], which is opposite to my finding in *T*. *dichotomus*. The sensory organ of CO_2_ is likely different even among species belonging to the same family (e.g., Scarabaeidae).

In some insect species, individuals aggregate through pheromone or other signals to gain benefits such as predation avoidance, thermoregulation and efficient access to food [16]. In contrast, results of the choice test between synthetic CO_2_ and larvae indicate that cues other than respiratory CO_2_ play no or only a negligible role in conspecific attraction in *T*. *dichotomus*. Furthermore, the aggregation is unlikely to enhance larval growth. Conspecific attraction through respiratory CO_2_ in this species may be a by-product of the response to CO_2_ as a cue for food.

Larval respiration is not the only factor responsible for higher concentrations of CO_2_ near the vicinity of larvae. I showed that the presence of larvae led to an increase in soil respiration and soil temperature. This suggests that soil microbes were activated near the vicinity of larvae. Soil microbes have been reported to be activated through the feeding and excretion processes of various kinds of soil-living invertebrates [32–34]. Although the mechanism underlying the stimulation of microbes by *T*. *dichotomus* larvae is unclear, the larvae probably generate a CO_2_ gradient both by their own respiration and by activating soil microbes.

In summary, larvae of *T*. *dichotomus* are attracted to CO_2_. Because their food emits CO_2_, it is probably a critical cue for food in the soil-living larvae. The clumped distribution of larvae within microhabitats reported in a previous study [11] is likely formed along the concentration gradient of CO_2_ created by heterogeneous saprophytic activities in soil. In addition, I found that larvae are attracted to respiratory CO_2_ of neighbours. Not only response to fermented food resources, but also conspecific attraction may result in aggregation. When larvae aggregate at a zone of high saprophytic activities, they compete for food and/or space, reducing larval growth gain. However, the cost of competition was outweighed by the benefit for exploiting high-quality food resources, and thus the response to CO_2_ is adaptive in this species.
